# Association of infrarenal aortic diameter in siblings within the Stockholm abdominal aortic aneurysm screening programme

**DOI:** 10.1093/bjs/znag108

**Published:** 2026-09-10

**Authors:** Antti Siika, Rebecka Hultgren, Jonas Brinck, Daniel P Andersson

**Affiliations:** Department of Molecular Medicine and Surgery, Karolinska Institutet, Stockholm, Sweden; Department of Molecular Medicine and Surgery, Karolinska Institutet, Stockholm, Sweden; Department of Vascular Surgery, Karolinska University Hospital, Stockholm, Sweden; Department of Medicine-H7 Huddinge, Karolinska Institutet, Stockholm, Sweden; Department of Endocrinology, Karolinska University Hospital Huddinge, Stockholm, Sweden; Department of Medicine-H7 Huddinge, Karolinska Institutet, Stockholm, Sweden; Department of Endocrinology, Karolinska University Hospital Huddinge, Stockholm, Sweden

## Abstract

**Background:**

The aim was to investigate the increased risk for siblings to have similar aortic pathology (wide or small) in an abdominal aortic aneurysm (AAA) screening population with ultrasonographic measurement of infrarenal aortic diameter (IAD).

**Methods:**

The population-based cohort of men who had participated in the Stockholm AAA screening programme between 2010 and 2024 (107 336 men) were linked to the Swedish Multigeneration Registry (MGR), via the STRIREG cohort. All individuals except one had data available in the MGR. A subaneurysmal aorta (SAA) was defined as having an IAD of 25–29 mm, an AAA was defined as having an IAD of ≥30 mm, and a small aorta was defined as having an IAD of <17 mm. Descriptive statistics and cumulative probability models were stratified for relatedness. Quantitative genetic modelling was used to estimate heritability.

**Results:**

From 16 005 screened 65-year-old men, 8987 sibling pairs (7625 pairs of full siblings, 1152 pairs of half siblings, and 210 pairs of twins) were included. In total, 161 (1.0%) had an AAA, 155 (1.0%) had an SAA, and 2700 (16.9%) had a small aorta. IAD correlated stronger in twins than full siblings and weakest among half siblings (half siblings, r = 0.1; full siblings, r = 0.19; and twins, r = 0.52). Overall, 316 (2.0%) individuals had an IAD ≥25 mm and, if a sibling’s IAD was 30 mm, the estimated probability of IAD ≥25 mm was 2.7% (95% c.i. 1.9% to 3.7%) in half siblings, 5.3% (95% c.i. 4.5% to 6.2%) in full siblings, and 21% (95% c.i. 10.2% to 38.2%) in twins. Heritability estimates were 0.41 for IAD, 0.61 for SAA/AAA, and 0.58 for small aorta.

**Conclusion:**

This study provides further evidence for familial aggregation and heritability of IAD and pathologies of the infrarenal aorta, which reinforces the advice for screening of siblings, and especially twins, of individuals with an abnormal aortic diameter including subaneurysms.

## Teaser

A high heritability of infrarenal aortic diameter and aortic pathologies, both widening and narrowing, is shown in this population-based study with complete case ascertainment based on infrarenal aortic diameter measurements from an abdominal aortic aneurysm screening programme.

## Introduction

The infrarenal aortic diameter (IAD) in the general population dilates with age^1^, but only a small proportion develop an aneurysmal pathology. All aortic pathologies (small aorta, but especially larger) in men diagnosed in the population-based screening programme confer an increased risk of mortality^2–4^.

Current guidelines recommend screening for abdominal aortic aneurysm (AAA) in individuals aged ≥50 years who have a first-degree relative with a known AAA^5^. Previous studies have shown that the IAD is larger in those with a family history of AAA^6^ and that up to a third of those with an AAA have an aortic enlargement^7^. Twin studies have demonstrated a high heritability of AAA, estimated at 70–77%^8,9^. A recent meta-analysis that synthesized results from a large number of studies estimated that the risk of AAA in a first-degree relative was increased by more than three-fold^10^. However, a key limitation in many studies on familial clustering, due to the disease’s asymptomatic nature, is the limited availability of data on aortic diameter in both affected and unaffected individuals^10^. Consequently, the familial aggregation of subaneurysmal dilations, small aortas, or normal aortic diameters remains insufficiently characterized.

In Sweden, a population-based screening programme exists where all 65-year-old men are invited to a one-time ultrasonographic screening^11,12^. Participation is high, with an attendance rate of approximately 80%^2^. Through linkage with national population registries, this framework enables investigation of familial clustering of aortic diameters among siblings. The aims of this study were to investigate the familial aggregation and heritability among siblings of four infrarenal aortic phenotypes (IAD, AAA, subaneurysmal widening, and small aorta) and to calculate the risk of having a pathological widening or narrowing depending on observed conditions in siblings.

## Methods

### Cohort

The cohort of men who had participated in the Stockholm AAA screening programme between 2010 and 2024 (107 336 men) were linked to the Swedish Multigeneration Registry (MGR)^13^, via the Stockholm hyperTRIglyceridemia REGister (STRIREG) study^14^. The study was approved by the Swedish Ethical Review Authority (2021-03883, 202408453-02, and 2022-02217-02).

All individuals except one had data available in the MGR. There was missing information on the mother for 21 679 individuals (20%) and on the father for 24 282 individuals (23%). The missing data were strongly related to registered birth country; among those born in Sweden (82 953 individuals), there was no information on the mother for 645 individuals (0.8%) and no information on the father for 2711 individuals (3.3%). Full siblings born on the same (or following) day were considered twins. No data on twin zygosity were available.

All persons who had participated in the screening programme and had at least one known sibling who had also participated in the screening programme (called sibling pairs), full/half (including those with one unknown parent) were included. Thus, individuals could be part of several sibling pairs.

Four different phenotypes were considered regarding the IAD, which is measured with the leading edge to leading edge ultrasonographic method in the Swedish screening programme^11^. An AAA was defined as having an IAD of ≥30 mm and a subaneurysmal aorta (SAA) was defined as having an IAD of 25–29 mm. Most SAAs progress to AAAs given time and, consequently, are likely to represent an earlier disease stage^15,16^. The inclusive phenotype of SAA/AAA (IAD ≥25 mm) was therefore considered. A small aorta was defined as having an IAD of <17 mm.

### Statistical analysis

For the continuous phenotype IAD, correlation between sibling pairs was estimated with generalized estimating equations implemented in the R package ‘drgee’^17^, in separate models by relatedness. Infrarenal diameter was log-transformed, due to an assumed lognormal distribution for the main analysis, but is also shown with untransformed axes.

A cumulative probability model, which models the cumulative distribution of an outcome and allows for estimation of the probability of exceeding a specified outcome value, as well as the median and percentiles of the outcome, was used (implemented in the R package ‘rms’^18^). The model used a logistic link and included an interaction between sibling relatedness in the pairs and infrarenal diameter, which was allowed to vary non-linearly with a restricted spline with three knots placed at the 10th, 50th, and 90th percentiles. The model estimated the probability of exceeding a certain infrarenal diameter threshold. Robust standard errors for clusters of sibling pairs by Huber-White were used.

To estimate influence of a potential shared environmental between full siblings that would contribute to the phenotypes, a cumulative probability model that included infrarenal diameter of the sibling moderated by time between birth of the sibling pair was used. Time between siblings was included as a continuous variable and was included with a restricted cubic spline with three knots.

To estimate familial aggregation for the binary phenotypes, AAA, SAA/AAA, and small aorta, concordance, tetrachoric correlation, and aggregation were computed. Concordance corresponds to the proportion of individuals who have the phenotype if the paired sibling has the phenotype and aggregation corresponds to the OR of having the phenotype depending on the phenotype of the sibling. Both concordance and aggregation were estimated with generalized estimating equations implemented in the *R* package ‘drgee’, allowing for standard errors to be corrected for non-independence of observations in sibling pairs^17^. Tetrachoric correlation assumes that the observed binary values represent the realization of a liability threshold of two underlying unobserved continuous Gaussian variables, and aims to estimate the correlation between the underlying variables, as implemented in the R package ‘polycor’^19^. For aggregation, concordance, and tetrachoric correlation, models were fit separately for relatedness.

To estimate the relative contributions of genetic and environmental factors to variation in the different phenotypes, ‘quantitative genetic analysis’ was used^20^. This type of analysis decomposes the phenotypic variance into additive genetic effects (A), shared common environment effects (C), and individual environment effects (E) across pairs of siblings and half siblings. This analysis was performed with Structural Equation Models in OpenMx^21^. The analysis is similar to the classical biometric twin model, but was modified to assume a shared genetic correlation of 0.5 in full siblings (including twins) and 0.25 in half siblings. In short, the total phenotypic variance is assumed to be determined by latent variables that correspond to A, C, and E, and a correlation corresponding to the genetic correlation is assumed for A. C represents shared environmental effects, which are non-genetic effects shared by the sibling pairs. No information on cohabitation during youth was available and this was assumed to be equal for siblings and half siblings. E represents individual environmental factors. Models were also fitted without the C term and, in all cases, the AE model did not fit the data inferiorly, as assessed by a likelihood ratio test. Due to a limited sample size and assumed similarity between the phenotype AAA and SAA or AAA, only the latter was retained for the quantitative genetic analysis.

All analyses were performed with the R Statistical Programming Language, version 4.6.0^22^.

## Results

All participants were 65-year-old men at the time of screening. The median infrarenal diameter among the participants was 18 (interquartile range 17–20) mm. Overall, 161 (1.0%) had an AAA, 155 (1.0%) had an SAA, and 2700 (16.9%) had a small aorta. In total, 8987 unique pairs (7625 pairs of full siblings, 1152 pairs of half siblings, and 210 pairs of twins) from 16 005 individuals for whom IAD had been measured in the screening programme were included (*Table 1*).

**Table 1:** Patient cohort

Unique individuals, *n*	16 005
Unique pairs, *n*	8987
**Outcomes**	
IAD (mm), median (interquartile range)	18 (17–20)
AAA	161 (1.01)
SAA	155 (0.97)
Small aorta	2700 (16.9)
**Sibling pairs**	
Full siblings	7625 (84.8)
Half siblings	1152 (12.8)
Twins	210 (2.3)

Values are *n* (%) unless otherwise indicated. IAD, infrarenal aortic diameter; AAA, abdominal aortic aneurysm; SAA, subaneurysmal aorta.

**Figure 1:**
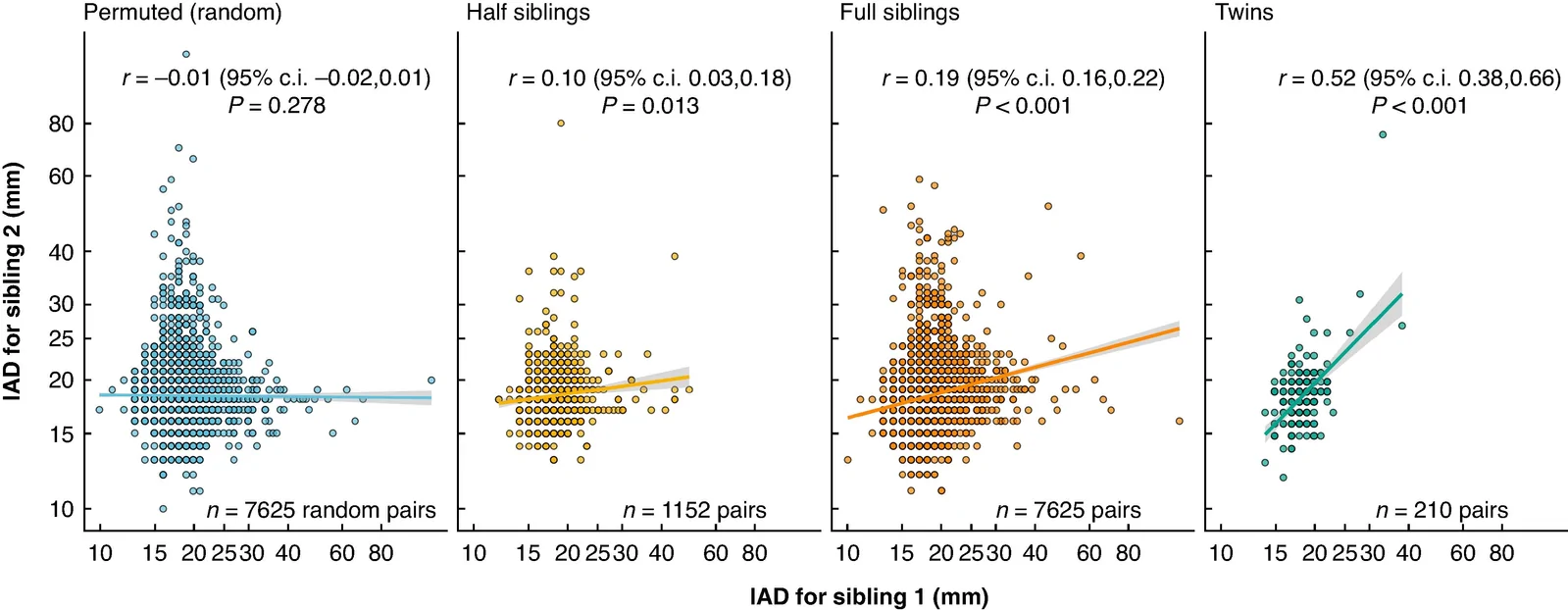
Correlation of infrarenal aortic diameter between related individuals. Estimates from generalized estimating equations with log-transformed IAD, accounting for clustering in sibling pairs. A single observation per pair is plotted. IAD, infrarenal aortic diameter.

Correlation between sibling pairs was assessed, stratified by relatedness. The correlation between half siblings was modest (r = 0.1 (95% c.i. 0.03 to 0.18)), higher among full siblings (r = 0.19 (95% c.i. 0.16 to 0.22)), and strongest among twins (r = 0.52 (95% c.i. 0.38 to 0.66)) (*Fig. 1*). Estimates for an untransformed diameter, and when excluding IADs ≥35 mm, were similar (*Fig. S1*).

### Cumulative probability of infrarenal aortic widening or narrowing

**Figure 2:**
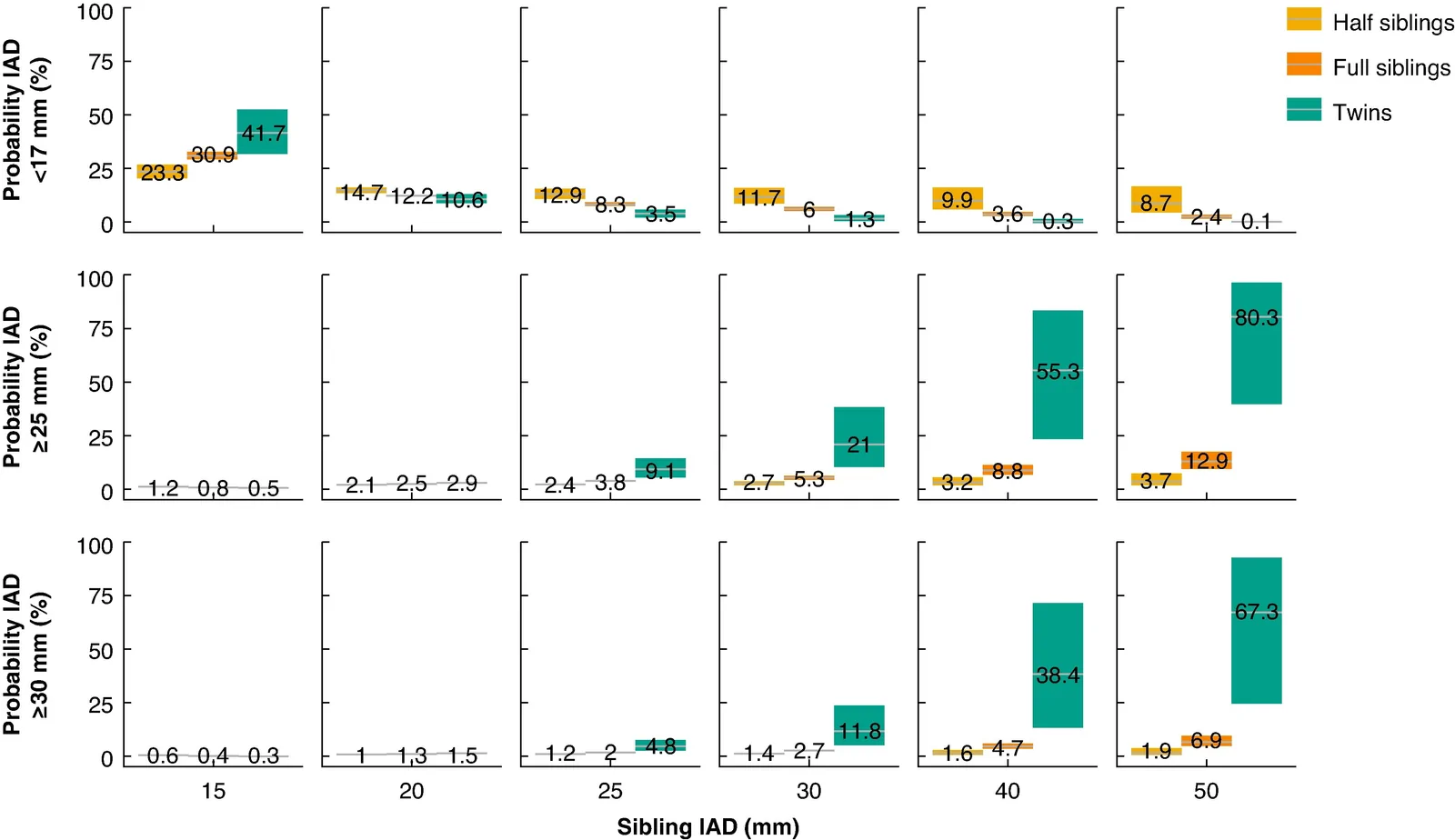
Estimated probabilities (with 95% confidence intervals) that a sibling has an IAD <17, ≥25, or ≥30 mm. Estimates represent predictions from a cumulative probability model with IAD as outcome, and sibling IAD and relatedness, and their interaction, as covariates. IAD, infrarenal aortic diameter.

Further, the cumulative probability of having an infrarenal aortic widening or narrowing was estimated. The interaction between relatedness was significant, where twins and full siblings had a higher risk of having a widening when the sibling had a widening, and the opposite when the sibling had a smaller diameter (*Fig. 2* and *Table S1*). The probability of having an IAD ≥25 mm was 2.7% (95% c.i. 1.9% to 3.7%) for an individual with a half sibling with an IAD of 30 mm, 5.3% (95% c.i. 4.5% to 6.2%) for an individual with a full sibling with an IAD of 30 mm, and 21% (95% c.i. 10.2% to 38.2%) for an individual with a twin with an IAD of 30 mm (*Table S1*). The probability of having an AAA (IAD ≥30 mm) was 1.2% (95% c.i. 1.5% to 1%) in half siblings, 2% (1.8% to 2.2%) in full siblings, and 4.8% (95% c.i. 2.9% to 7.7%) in twins whose sibling had an IAD of 25 mm. Expected percentiles of IAD according to sibling IAD and relatedness are shown in *Fig. S2*.

To assess whether the familial aggregation across siblings included shared non-genetic effects, percentiles of IAD in full siblings, moderated by birth year gap between the siblings, was estimated. The interaction between sibling diameter and gap in birth years was significant (*P* = 0.0323), and the predicted percentiles of IAD, especially in the highest percentiles was attenuated as the gap in birth years increased (*Fig. S3*).

### Concordance, coaggregation, and correlation

Measures for association for the binary phenotypes of AAA, SAA/AAA, and small aorta are presented in *Table 2*. Full siblings who had a sibling with an AAA had an OR of 5.69 (95% c.i. 2.03 to 15.98) of having an AAA and full siblings who had a sibling with an SAA/AAA had an OR of 3.44 of having an SAA/AAA. Twins had an OR of 91.56 (95% c.i. 4.54 to 1846.62) of having an AAA if they had a twin with an AAA and an OR of 202 (95% c.i. 22.4 to 1824.7) of having an SAA/AAA if they had a twin with an SAA/AAA (*Table 2*). Among half siblings with both known parents in the MGR, there were no concordant cases for either AAA or SAA/AAA, but, including those with one unknown parent, the OR for having an AAA was 7.83 (95% c.i. 0.94 to 65.12), which was higher than for full siblings, but with a wide confidence interval overlapping 1; including those with one unknown parent, the OR for having an SAA/AAA was 1.76 (95% c.i. 0.23 to 13.53).

**Table 2:** Descriptive statistics of similarity between half siblings, full siblings, and twins for AAA, SAA/AAA, and small aorta

	Total pairs	Concordant pairs (−/−)	Concordant pairs (+/+)	Discordant pairs (+/−)	Concordance rate (95% c.i.)	Tetrachoriccorrelation (95% c.i.)	Aggregation OR (95% c.i.)
**AAA (IAD ≥30 mm)**							
Half siblings	1152	1127	1	24	0.08 (−0.07,0.22)	0.36 (0.06,0.66)	7.83 (0.94,65.12)
Half siblings (both known parents)	788	771	0	17	0 (0,0)	-	0 (0,0)
Full siblings	7625	7476	4	145	0.05 (0,0.1)	0.29 (0.15,0.43)	5.69 (2.03,15.98)
Twins	210	206	1	3	0.4 (−0.23,1.03)	0.81 (0.53,1.08)	91.56 (4.54,1846.62)
**SAA/AAA (IAD ≥25 mm)**							
Half siblings	1152	1101	1	50	0.04 (−0.04,0.11)	0.1 (−0.17,0.38)	1.76 (0.23,13.53)
Half siblings (both known parents)	788	752	0	36	0 (0,0)	-	0 (0,0)
Full siblings	7625	7339	9	277	0.06 (0.02,0.1)	0.23 (0.13,0.33)	3.44 (1.72,6.90)
Twins	210	202	4	4	0.67 (0.34,1)	0.93 (0.84,1.02)	202.00 (22.36,1824.67)
**Small aorta (IAD <17 mm)**							
Half siblings	1152	790	42	320	0.21 (0.15,0.26)	0.08 (0,0.17)	1.30 (0.89,1.90)
Half siblings (both known parents)	788	539	32	217	0.23 (0.16,0.29)	0.12 (0.02,0.23)	1.47 (0.94,2.29)
Full siblings	7625	5455	369	1801	0.29 (0.27,0.31)	0.29 (0.26,0.32)	2.48 (2.16,2.86)
Twins	210	149	12	49	0.33 (0.19,0.47)	0.35 (0.17,0.53)	2.98 (1.32,6.72)

Both known parents includes only half siblings where both parents are known in the MGR. AAA, abdominal aortic aneurysm; SAA, subaneurysmal aorta; IAD, infrarenal aortic diameter; MGR, Multigeneration Registry.

### Quantitative genetic analysis

To specifically estimate the heritability contribution to the phenotypes, a quantitative genetic analysis was performed among full siblings and half siblings. This showed that IAD had a heritability of 0.41, SAA/AAA had a heritability of 0.61, and small aorta had a heritability of 0.58, all with no estimated contribution from a shared environment (*Table 3*).

**Table 3:** Estimation of heritability and environmental influences on IAD, AAA/SAA, and small aorta using univariate ACE/AE models in full siblings and half siblings

Trait and type	h2 (95% c.i.)	c2 (95% c.i.)	e2 (95% c.i.)
**IAD**			
ACE	0.41 (0.37,0.45)	0.00 (0.00,0.00)	0.59 (0.55,0.63)
AE	0.41 (0.37,0.45)		0.59 (0.55,0.63)
**SAA/AAA (IAD ≥25 mm)**			
ACE	0.61 (0.36,0.86)	0.00 (0.00,0.00)	0.39 (0.14,0.64)
AE	0.61 (0.36,0.86)		0.39 (0.14,0.64)
**Small aorta (IAD <17 mm)**			
ACE	0.58 (0.49,0.66)	0.00 (0.00,0.00)	0.42 (0.34,0.51)
AE	0.58 (0.49,0.66)		0.42 (0.34,0.51)

IAD, infrarenal aortic diameter; AAA, abdominal aortic aneurysm; SAA, subaneurysmal aorta; h2, heritability (genetic variance); c2, shared environmental variance; e2, unique environmental variance; ACE, full biometric model; AE, reduced biometric model without shared environmental variance between siblings.

## Discussion

The high heritability of infrarenal aortic pathologies, as previously suggested by family-based studies, is confirmed in a large, homogeneous, population-based AAA screening programme with IAD measurements. Consequently, these results support the societal recommendations for siblings of individuals with an AAA to be screened, but also suggests an increased risk for relatives with subaneurysmal diameters.

Heritability of aortic diameter among men has previously been estimated at 0.42, adjusted for age, in a family-based study^23^, which is almost equivalent to what is found in the present study. As a comparison, studies of human height have shown considerably higher heritability of 0.8–0.9^24,25^. While both represent anthropometric variables, the discrepancy may potentially be explained by a greater influence of environment on the phenotype of infrarenal aorta, which may be more similar to body weight, which is also more influenced by environmental factors compared with height^26^. A similar pattern to what has been observed for weight, that is the heritability of obesity is higher than that for weight in general, is also observed in the present study^27^. Pathological phenotypes appear to have higher heritability than IAD in general. A recent genome-wide association study (GWAS) estimated single-nucleotide polymorphism (SNP)-based heritability for IAD of 0.2^28^, which may indicate that current GWASs for IAD are still underpowered to detect all relevant genetic variants.

The comparatively high correlation between twins for the aneurysmal phenotypes and infrarenal diameter, even compared with full siblings, and the attenuated correlation between IAD with increasing gap of birth year may be suggestive of a shared environmental factor. While the quantitative genetic analysis should be interpreted with caution, as limited information was available to estimate an expected contribution of shared environmental factors in the sibling pairs (for example no information on cohabitation during childhood or later relationship was available), this showed no role of a shared environment. The absence of a shared environmental effect is consistent with findings from previous twin studies, which have shown no significant influence of a shared environment on the AAA phenotype^8,9^. Non-genetic familial aggregation could conceivably be caused by, for example, smoking behaviour, which has been shown to some extent to have shared familial non-genetic causes and is a strong risk factor for AAA^29^. Together, this likely points to an underestimation of shared environmental effects and may contribute to an overestimation of genetic effects.

Persons will small aortic diameters have historically had a low degree of attention in the literature and in practical clinical care. As the proportion of such men is not that small (15–20% aged 65 years) and such men have a significant increased risk of premature death^2–4^, further targeted attempts to characterize this group are necessary. It is probable that there is a strong association between smaller aortic diameters and a cardiovascular risk profile, which needs to be explored further in a contemporary setting.

No analysis including twin zygosity could be undertaken, as this was not known from the registry. A low sample size for the SAA/AAA phenotype among siblings and half siblings resulted in a wide confidence interval and uncertainty in quantitative genetic models for this phenotype and limits the generalizability. The inclusive phenotype of SAA and AAA should conceivably capture all relevant heritability, as they likely represent the same pathology. The true effect of a shared environment is likely difficult to fully capture as many influential environmental factors may occur late in life, whereas family structure is more likely to reflect a shared environment in early life.

One limitation of the analysis is the inclusion of only men, as only men are invited to the AAA screening programme. Even with an almost complete coverage of the regional population of men aged 65 years, the actual numbers of diagnosed patients are still quite low. An investigation of these risk associations in a cohort of women with aortic disease would require another international multicentre database design, in order to obtain sufficient sample sizes. It is, however, plausible that these findings are quite applicable also for women with risk for disease. Further, only siblings who attended the AAA screening were included, which limits a full analysis of clustering in families and may exclude high-risk families that are examined outside of the screening programme. A major risk factor for aortic disease, smoking, was not collected within the full screening programme and could therefore not be analysed. The nature of the screening programme with invited men and all examined at the same age, however, implicitly adjusts for two major confounders of aortic diameter, that is age and sex, and importantly the phenotype of all participating individuals is ascertained.

## Supplementary Material

znag108_Supplementary_Data

## Data Availability

The data that support the findings of this study are not publicly available. Access to data might be granted after review by the STRIREG steering committee.

## References

[1] Obel LM, Diederichsen AC, Steffensen FH, Frost L, Lambrechtsen J, Busk M et al Population-based risk factors for ascending, arch, descending, and abdominal aortic dilations for 60-74-year-old individuals. J Am Coll Cardiol 2021;78:201–211 34266574 10.1016/j.jacc.2021.04.094

[2] Siika A, Axelsson A, Fattahi N, Roy J, Öhman D, Linné A et al Decreasing aortic diameter and decreasing prevalence of infrarenal aortic aneurysms in a population-based screening programme. Br J Surg 2025;112:znaf156 40834047 10.1093/bjs/znaf156 PMC12366718

[3] Norman PE, Muller J, Golledge J. The cardiovascular and prognostic significance of the infrarenal aortic diameter. J Vasc Surg 2011;54:1817–1820 21944921 10.1016/j.jvs.2011.07.048

[4] Sidloff DA, Saratzis A, Thompson J, Katsogridakis E, Bown MJ. Editor’s Choice—Infra-renal aortic diameter and cardiovascular risk: making better use of abdominal aortic aneurysm screening outcomes. Eur J Vasc Endovasc Surg 2021;62:38–45 33985908 10.1016/j.ejvs.2021.03.013

[5] Wanhainen A, Van Herzeele I, Bastos Goncalves F, Bellmunt Montoya S, Berard X, Boyle JR et al Editor’s Choice—European Society for Vascular Surgery (ESVS) 2024 clinical practice guidelines on the management of abdominal aorto-iliac artery aneurysms. Eur J Vasc Endovasc Surg 2024;67:192–331 38307694 10.1016/j.ejvs.2023.11.002

[6] Joergensen TMM, Houlind K, Green A, Lindholt JS. Abdominal aortic diameter is increased in males with a family history of abdominal aortic aneurysms: results from the Danish VIVA-trial. Eur J Vasc Endovasc Surg 2014;48:669–675 25443525 10.1016/j.ejvs.2014.09.005

[7] Frydman G, Walker PJ, Summers K, West M, Xu D, Lightfoot T et al The value of screening in siblings of patients with abdominal aortic aneurysm. Eur J Vasc Endovasc Surg 2003;26:396–400 14512002 10.1016/s1078-5884(03)00316-2

[8] Wahlgren CM, Larsson E, Magnusson PKE, Hultgren R, Swedenborg J. Genetic and environmental contributions to abdominal aortic aneurysm development in a twin population. J Vasc Surg 2010;51:3–7 19939604 10.1016/j.jvs.2009.08.036

[9] Joergensen TMM, Christensen K, Lindholt JS, Larsen LA, Green A, Houlind K. Editor’s Choice—High heritability of liability to abdominal aortic aneurysms: a population based twin study. Eur J Vasc Endovasc Surg 2016;52:41–46 27107486 10.1016/j.ejvs.2016.03.012

[10] Fattahi N, Stenman M, Hultgren R. Editor’s Choice—The prevalence of abdominal aortic aneurysm in first degree relatives: a systematic review and meta-analysis. Eur J Vasc Endovasc Surg 2026;71:540–551 41456790 10.1016/j.ejvs.2025.12.044

[11] Wanhainen A, Hultgren R, Linné A, Holst J, Gottsäter A, Langenskiöld M et al Outcome of the Swedish nationwide abdominal aortic aneurysm screening program. Circulation 2016;134:1141–1148 27630132 10.1161/CIRCULATIONAHA.116.022305

[12] Earnshaw JJ, Mitra S, Strachan H, Gardner P; Effectiveness Review Collaborators. Abdominal aortic aneurysm screening: current effectiveness and future perspectives. Br J Surg 2025;112:znaf094 40328447 10.1093/bjs/znaf094

[13] Ekbom A . The Swedish Multi-generation Register. Methods Mol Biol 2011;675:215–220 20949391 10.1007/978-1-59745-423-0_10

[14] Andersson DP, Littmann K, Kindborg G, Eklund D, Sejersen K, Yan J et al Relation among hypertriglyceridaemia, cardiometabolic disease, and hereditary factors—design and rationale of the Stockholm hyperTRIglyceridaemia REGister study. Eur Heart J Open 2024;4:oeae010 38487365 10.1093/ehjopen/oeae010 PMC10937219

[15] Thorbjørnsen K, Svensjö S, Gilgen NP, Wanhainen A. Long term outcome of screen detected sub-aneurysmal aortas in 65 year old men: a single scan after five years identifies those at risk of needing AAA repair. Eur J Vasc Endovasc Surg 2021;62:380–386 34362628 10.1016/j.ejvs.2021.05.039

[16] Wild JB, Stather PW, Biancari F, Choke EC, Earnshaw JJ, Grant SW et al A multicentre observational study of the outcomes of screening detected sub-aneurysmal aortic dilatation. Eur J Vasc Endovasc Surg 2013;45:128–134 23273900 10.1016/j.ejvs.2012.11.024

[17] Zetterqvist J, Sjölander A. Doubly robust estimation with the R package drgee. Epidemiol Methods 2015;4:69–86

[18] Liu Q, Shepherd BE, Li C, Harrell FE Jr. Modeling continuous response variables using ordinal regression. Stat Med 2017;36:4316–4335 28872693 10.1002/sim.7433 PMC5675816

[19] Fox J, Dusa A, Murdoch D. *polycor: Polychoric and Polyserial Correlations*. 2025. https://cran.r-project.org/web/packages/polycor/index.html (accessed 8 January 2026)

[20] Neale MC, Maes HHM. Methodology for Genetic Studies of Twins and Families. B.V. Dordrecht, The Netherlands: Kluwer Academic Publishers, 2004

[21] Boker SM, Neale MC, Maes HH, Wilde MJ, Spiegel M, Brick TR et al *OpenMx: Extended Structural Equation Modelling*. 2025. https://cran.r-project.org/web/packages/OpenMx/index.html (accessed 9 January 2026)

[22] R Core Team . R: A Language and Environment for Statistical Computing. Vienna, Austria: R Foundation for Statistical Computing, 2025. https://www.R-project.org/

[23] Peypoch O, Paüls-Vergés F, Vázquez-Santiago M, Dilme J, Romero J, Giner J et al The TAGA study: a study of factors determining aortic diameter in families at high risk of abdominal aortic aneurysm reveal two new candidate genes. J Clin Med 2020;9:1242 32344696 10.3390/jcm9041242 PMC7231034

[24] Conery M, Grant SFA. Human height: a model common complex trait. Ann Hum Biol 2023;50:258–266 37343163 10.1080/03014460.2023.2215546 PMC10368389

[25] Jelenkovic A, Sund R, Hur Y-M, Yokoyama Y, Hjelmborg JVB, Möller S et al Genetic and environmental influences on height from infancy to early adulthood: an individual-based pooled analysis of 45 twin cohorts. Sci Rep 2016;6:28496 27333805 10.1038/srep28496 PMC4917845

[26] Chodick G, Simchoni M, Jensen BW, Derazne E, Pinhas-Hamiel O, Landau R et al Heritability of body mass index among familial generations. JAMA Netw Open 2024;7:e2419029 38941093 10.1001/jamanetworkopen.2024.19029 PMC11214117

[27] Bouchard C . Genetics of obesity: what we have learned over decades of research. Obesity 2021;29:802–820 33899337 10.1002/oby.23116

[28] Portilla-Fernandez E, Klarin D, Hwang S-J, Biggs ML, Bis JC, Weiss S et al Genetic and clinical determinants of abdominal aortic diameter: genome-wide association studies, exome array data and Mendelian randomization study. Hum Mol Genet 2022;31:3566–3579 35234888 10.1093/hmg/ddac051 PMC9558840

[29] Vink JM, Boomsma DI. Interplay between heritability of smoking and environmental conditions? A comparison of two birth cohorts. BMC Public Health 2011;11:316 21569578 10.1186/1471-2458-11-316 PMC3112130

