# Supplementary material for: Association of infrarenal aortic diameter in siblings within the Stockholm abdominal aortic aneurysm screening programme
Source: Br J Surg. 2026 Sep 10;113(9):znag108. doi: 10.1093/bjs/znag108 (PMC13560079; doi:10.1093/bjs/znag108)
Supplement: Supplementary file 1 [file znag108_supplementary_data.docx]

**Supplementary Material:**

Association of Infrarenal Aortic Diameter in Siblings within the Stockholm Abdominal Aortic Aneurysm Screening Programme

Antti Siika (1), Rebecka Hultgren (1,2), Jonas Brinck (3,4), Daniel P Andersson (3,4)

(1) Department of Molecular Medicine and Surgery, Karolinska Institutet, Stockholm Sweden
(2) Department of Vascular Surgery, Karolinska University Hospital, Stockholm, Sweden
(3) Department of Medicine-H7 Huddinge, Karolinska Institutet, Stockholm, Sweden
(4) Department of Endocrinology, Karolinska University Hospital Huddinge, Stockholm, Sweden

List of Tables and Figures

[Supplementary Table S1. Estimated probabilities (with 95% CI) that a sibling has an infrarenal aortic diameter (IAD) exceeding specified thresholds given the IAD of the index sibling	2](#_Toc222907885)

[Supplementary Figure S1. Estimated probabilities (with 95% CI) that a sibling has an infrarenal aortic diameter (IAD) exceeding specified thresholds given the IAD of the index sibling, stratified by zygosity. (A). Untransformed IAD, (B), IAD < 35 mm	4](#_Toc222907886)

[Supplementary Figure S2. Estimated quantiles of infrarenal aortic diameter (IAD) in siblings given the IAD of the index sibling, stratified by relatedness.	5](#_Toc222907887)

[Supplementary Figure S3. IAD by diameter in full siblings, by year between birth years. Note that gap between birthyears was modeled as a continuous variable and is shown for three different values	6](#_Toc222907888)

| Supplementary Table S1. Estimated probabilities (with 95% CI) that a sibling has an infrarenal aortic diameter (IAD) exceeding specified thresholds given the IAD of the index sibling**.** | | | |
| --- | --- | --- | --- |
| Sibling IAD (mm) | Prob IAD < 17 mm | Prob IAD ≥ 25 mm | Prob IAD ≥ 30 mm |
| 15 |  |  |  |
| Twins | 41.7% (31.7%-52.3%) | 0.5% (0.3%-0.8%) | 0.3% (0.2%-0.4%) |
| Full siblings | 30.9% (29.3%-32.6%) | 0.8% (0.7%-0.9%) | 0.4% (0.4%-0.4%) |
| Half-siblings | 23.3% (20.1%-26.7%) | 1.2% (1%-1.4%) | 0.6% (0.5%-0.7%) |
| 18 |  |  |  |
| Twins | 17.8% (15.1% -20.9%) | 1.6% (1.3% -2%) | 0.8% (0.7%-1%) |
| Full siblings | 15.7% (15.2% -16.2%) | 1.9% (1.8% -2%) | 1% (0.9%-1%) |
| Half-siblings | 16.3% (15.1% -17.5%) | 1.8% (1.7% -2%) | 0.9% (0.8%-1%) |
| 20 |  |  |  |
| Twins | 10.6% (8.8% -12.8%) | 2.9% (2.4% -3.6%) | 1.5% (1.2%-1.9%) |
| Full siblings | 12.2% (11.8% -12.7%) | 2.5% (2.4% -2.6%) | 1.3% (1.2%-1.3%) |
| Half-siblings | 14.7% (13.6% -15.9%) | 2.1% (1.9% -2.2%) | 1% (1%-1.1%) |
| 25 |  |  |  |
| Twins | 3.5% (2.1%-5.6%) | 9.1% (5.7%-14.3%) | 4.8% (2.9%-7.7%) |
| Full siblings | 8.3% (7.6%-9.1%) | 3.8% (3.5%-4.2%) | 2% (1.8%-2.2%) |
| Half-siblings | 12.9% (10.8%-15.4%) | 2.4% (1.9%-2.9%) | 1.2% (1%-1.5%) |
| 30 |  |  |  |
| Twins | 1.3% (0.6%-3.1%) | 21% (10.2%-38.2%) | 11.8% (5.4%-23.7%) |
| Full siblings | 6% (5.2%-7%) | 5.3% (4.5%-6.2%) | 2.7% (2.3%-3.2%) |
| Half-siblings | 11.7% (8.6%-15.6%) | 2.7% (1.9%-3.7%) | 1.4% (1%-1.9%) |
| 40 |  |  |  |
| Twins | 0.3% (0.1%-1.2%) | 55.3% (23.5%-83.3%) | 38.4% (13.4%-71.5%) |
| Full siblings | 3.6% (2.8%-4.6%) | 8.8% (6.9%-11.3%) | 4.7% (3.6%-6%) |
| Half-siblings | 9.9% (5.9%-16.1%) | 3.2% (1.8%-5.4%) | 1.6% (0.9%-2.8%) |
| 50 |  |  |  |
| Twins | 0.1% (0%-0.5%) | 80.3% (39.7%-96.2%) | 67.3% (24.9% -92.7%) |
| Full siblings | 2.4% (1.7%-3.3%) | 12.9% (9.5%-17.4%) | 6.9% (5%-9.6%) |
| Half-siblings | 8.7% (4.4%-16.5%) | 3.7% (1.8%-7.3%) | 1.9% (0.9%-3.8%) |
| IAD = infrarenal aortic diameter, CI = confidence interval | | | |

## Supplementary Figure S1. Estimated probabilities (with 95% CI) that a sibling has an infrarenal aortic diameter (IAD) exceeding specified thresholds given the IAD of the index sibling, stratified by zygosity. (A) *Untransformed* IAD, (B) Only individuals with in IAD < 35 mm

## Supplementary Figure S2. Estimated percentiles (median, 5^th^, 25^th^, 75^th^ and 95^th^) of infrarenal aortic diameter (IAD) given the IAD of the sibling. Separate panels show the result stratified by relatedness. Estimates are derived from a cumulative probability model with IAD as outcome, and sibling IAD and relatedness, and their interaction as covariates.

## Supplementary Figure S3. Median and percentiles of IAD, given an aortic diameter in a full sibling and the absolute gap between birth years of the full sibling pair. Note that gap between birth years was modeled as a continuous variable and is shown for three different gaps (1, 5 and 10 years).
